# Mesenchymal stem cells combined with autocrosslinked hyaluronic acid improve mouse ovarian function by activating the PI3K-AKT pathway in a paracrine manner

**DOI:** 10.1186/s13287-022-02724-3

**Published:** 2022-02-02

**Authors:** Wenlin Jiao, Xin Mi, Yajuan Yang, Ran Liu, Qiang Liu, Tao Yan, Zi-Jiang Chen, Yingying Qin, Shidou Zhao

**Affiliations:** 1 Center for Reproductive Medicine, Cheeloo College of Medicine, Shandong University, Jinan, 250012 Shandong China; 2 Key Laboratory of Reproductive Endocrinology of Ministry of Education, Shandong University, Jinan, 250012 Shandong China; 3 Shandong Key Laboratory of Reproductive Medicine, Jinan, 250012 Shandong China; 4 Shandong Provincial Clinical Research Center for Reproductive Health, Jinan, 250012 Shandong China; 5 National Research Center for Assisted Reproductive Technology and Reproductive Genetics, Shandong University, Jinan, 250012 Shandong China; 6 Shanghai Key Laboratory for Assisted Reproduction and Reproductive Genetics, Shanghai, 200135 China; 7 Center for Reproductive Medicine, School of Medicine, Ren Ji Hospital, Shanghai Jiao Tong University, 200135 Shanghai, China

**Keywords:** Mesenchymal stem cells, Hyaluronic acid, Paracrine, PI3K-AKT pathway, Premature ovarian insufficiency, Ovarian aging, Hepatocyte growth factor

## Abstract

**Background:**

Declining ovarian function in advance-aged women and in premature ovarian insufficiency (POI) patients seriously affects quality of life, and there is currently no effective treatment to rescue ovarian function in clinic. Stem cell transplantation is a promising therapeutic strategy for ovarian aging, but its clinical application is limited due to the low efficiency and unclear mechanism. Here, a novel combination of umbilical cord-mesenchymal stem cells (UC-MSCs) and autocrosslinked hyaluronic acid (HA) gel is explored to rescue ovarian reserve and fecundity in POI and naturally aging mice.

**Methods:**

To investigate HA prolonged the survival after UC-MSCs transplantation, PCR and immunofluorescence were performed to track the cells on day 1, 3, 7 and 14 after transplantation. The effects of HA on UC-MSCs were analyzed by CCK8 assay, RNA-sequencing and 440 cytokine array. In vivo experiments were conducted to evaluate the therapeutic effects of UC-MSCs combined with HA transplantation in 4-vinylcyclohexene diepoxide (VCD)-induced POI mice and naturally aging mice model. Ovarian function was analyzed by ovarian morphology, follicle counts, estrous cycle, hormone levels and fertility ability. To investigate the mechanisms of stem cell therapy, conditioned medium was collected from UC-MSCs and fibroblast. Both in vitro ovarian culture model and 440 cytokine array were applied to assess the paracrine effect and determine the underlying mechanism. Hepatocyte growth factor (HGF) was identified as an effective factor and verified by HGF cytokine/neutralization antibody supplementation into ovarian culture system.

**Results:**

HA not only prolongs the retention of UC-MSCs in the ovary, but also boosts their secretory function, and UC-MSCs promote follicular survival by activating the PI3K-AKT pathway through a paracrine mechanism both in vitro and in vivo. More importantly, HGF is identified as the key functional cytokine secreted by MSCs.

**Conclusions:**

The results show that HA is an excellent cell scaffold to improve the treatment efficiency of UC-MSCs for ovarian aging under both physiological and pathological conditions, and the therapeutic mechanism is through activation of the PI3K-AKT pathway via HGF. These findings will facilitate the clinical application of MSCs transplantation for ovarian disorders.

**Supplementary Information:**

The online version contains supplementary material available at 10.1186/s13287-022-02724-3.

## Background

Female fecundity decreases with chronological age, and the ovary exhibits aging-associated dysfunction in humans [1, 2]. Physiological demise of follicles and impaired oocyte quality result in infertility and ultimately in the cessation of ovarian function [3]. In terms of pathological conditions, premature ovarian insufficiency (POI) is a common reproductive disorder characterized by menstrual disturbances with elevated gonadotrophin levels and decreased estradiol levels before the age of 40 years, and POI affects 1–5% of women [4]. Nowadays, the therapeutic options for ovarian functional decline, either physiologically or pathologically, are limited. Therefore, developing effective strategies will be beneficial for the physical, mental, and reproductive health of these women.

Recently, mesenchymal stem cells (MSCs) administration has emerged as a novel approach for tissue regeneration and for treating disease [5]. It is well established that MSCs transplantation can ameliorate ovarian function in human POI patients and in aging mice, as indicated by restored menstrual/estrous cycles, increased follicle numbers, and fertility [6, 7]. However, poor engraftment efficiency, insufficient viability of transplanted cells, and unclear mechanisms of action have greatly hampered their application in the clinic [8].

Several strategies have been introduced to improve the effectiveness of MSCs, such as the use of sodium alginate-bioglass composite hydrogels and type I collagen as scaffolds during transplantation [9, 10]. However, commercialized products widely accepted in clinical practice are still lacking. Hyaluronic acid (HA), a natural component of extracellular matrices, has been proposed as an emerging biopolymer for various tissue engineering applications due to its nontoxic, biocompatible, and biodegradable qualities [6, 11]. After autocrosslinking, HA presents with a gel-like viscoelastic behavior, which can increase its residence time in the body [12]. Recently, autocrosslinked HA has been reported as a stem cell scaffold for endometrial regeneration in rhesus monkeys [13]. Therefore, its application as a cell scaffold during ovarian MSCs transplantation is anticipated.

By generating a multifunctional secretome, MSCs can stimulate proliferation, inhibit apoptosis, and improve the microenvironment in the ovary [14, 15]. The finely balanced phosphatidylinositol 3 kinase (PI3K)-protein kinase B (AKT) pathway in the mammalian ovary is essential for folliculogenesis, and insufficient activation of this pathway will impair the survival of primordial follicles leading to POI [16, 17]. Interestingly, it has been reported that MSCs promote the healing of gastric ulcers and skin injuries by activating the PI3K-AKT signaling pathway [18, 19]. Whether MSCs also exert protective effects on primordial follicles through activating the PI3K-AKT pathway remains to be determined.

In this study, we used a novel transplantation strategy by combining umbilical cord-mesenchymal stem cells (UC-MSCs) with autocrosslinked HA gel, which not only increased the local retention of stem cells in the ovary, but also augmented the MSCs’ paracrine function. The therapeutic effects of stem cell transplantation were tested in both a 4-vinylcyclohexene diepoxide (VCD)-induced POI mouse model and in naturally aged mice. Mechanistically, MSCs ameliorated ovarian function by activating the PI3K-AKT pathway through a paracrine mechanism. More importantly, hepatocyte growth factor (HGF) was first identified as the effective component secreted by MSCs to promote follicle survival.

## Methods

### Cell culture and characterization

Human UC-MSCs and fibroblasts were cultured in α-minimum essential medium (Gibco) and minimum essential medium (Gibco), respectively, supplemented with 5% ultra-advanced GRO (Helios) plus 1% penicillin/streptomycin, and the medium was changed every 48 h. The cell surface antigens, including CD90, CD107, CD73, CD34, CD31, CD45, and HLA-DR (Biolegend), were analyzed by flow cytometry using phycoerythrin (PE)-conjugated human monoclonal antibodies. Mouse IgG isotype was used as the negative control. The differentiation capability of UC-MSCs was assessed with an Osteogenic, Adipogenic and Chondrogenic Differentiation Kit (Cyagen). In this study, MSCs between passages 6 and 9 were used in all experiments.

### CCK8 assay

The effect of different concentrations of HA on UC-MSCs’ viability was determined with the CCK-8 assay. UC-MSCs were cultured in 96-well plates and treated with a gradient concentration of HA (0–0.5 mg/ml). After 0–5 days of treatment, 10 µl CCK8 reagent (Beyotime) was added to each well of the plate and incubated for 2 h in a 37 °C incubator. The absorbance values were measured at 450 nm with a microplate spectrophotometer (SpectraMax Plus).

### RNA-sequencing

UC-MSCs were cultured in control medium or medium containing 0.3 mg/ml HA for five days. Total RNA was extracted by TRIzol reagent (Invitrogen). Subsequently, the samples were sequenced on an Illumina Novaseq6000 genome analyzer platform. The RNA-seq procedure and the raw data from the reactions were processed by Xiuyue Biol (Jinan, China). Bioinformatics analysis was performed to identify differentially expressed genes meeting the requirements of *P*_adj_ < 0.05 and |log_2_FoldChange|≥ 1 between the two groups.

### DNA extraction and agarose gel electrophoresis

The mice in the MSC group were injected with 5 µl 1 × 10^5^ UC-MSCs directly into the ovaries [20], and the mice in the MSC + HA group were transplanted with the same amount of cells combined with 0.1–0.3 mg/ml autocrosslinked HA (Bioregen). In the hypoxia group the UC-MSCs were preconditioned in a 5% O_2_ incubator for 48 h and transplanted in combination with 0.3 mg/ml HA. The ovaries in each group were collected at 1, 7, and 14 days after transplantation. The genomic DNA was extracted from the ovaries and UC-MSCs with TIANamp Genomic DNA Kits (Tiangen) following the protocols recommended by the manufacturer. The DNA concentrations were measured by a spectrophotometer (NanoDrop, Thermo Scientific). The extracted DNA was used for PCR amplification of fragments of specific regions of the human and mouse *GAPDH* genes. The primers were as follows: human *GAPDH*, F: 5'-GCA CCC TAT GGA CAC GCT C-3', R: 5'-CCC ACA TCA CCC CTC TAC CTC-3', 297 bp; mouse *GAPDH*, F: 5'-GTC ACT ACC GAA GAA CAA CGA G-3', R: 5'-TGT GGG CTC CGA ACT GAT-3', 307 bp. The amplified DNA fragments were subjected to electrophoresis in 2% agarose gels to verify the expression of human/mouse *GAPDH* in the transplanted ovaries, and the gels were visualized under ultra-violet light. Ovaries without stem cell injection were used as negative controls.

### Chemicals

For intraperitoneal injection into mice, 4-vinylcyclohexene diepoxide (VCD, Sigma) was dissolved in normal saline (0.9% sodium chloride) with 0.1% DMSO (Sigma). For ovarian culture, VCD was diluted in DMEM/F12 (Gibco) with 0.1% DMSO and added to the culture system at a final concentration of 30 µM. The PI3K inhibitor LY294002 (Selleck) was dissolved in DMSO and added to ovarian cultures at a final concentration of 20 µM, which was optimal for cultured ovaries and without toxic effects [21].

### Preparation of the MSC-CM and Fib-CM

UC-MSCs and fibroblasts were seeded separately at a density of 1 × 10^6^ in T75 flasks and cultured until the cells reached ~ 90% confluence. The culture medium was then removed and the cells were washed with PBS three times. The medium was changed to 12 mL Dulbecco's Modified Eagle Medium/Nutrient Mixture F-12 (DMEM/F12) (Gibco) media, and after culturing for 48 h the conditioned medium was collected and centrifuged at 1500 × *g* for 5 min to remove cell debris and then filtered through a 0.22 μm filter (Millipore). Finally, a 3 kDa cutoff centrifugal filter unit (Millipore) was used to concentrate the medium 25-fold by centrifugation at 5000 × *g* for 40–50 min, and the medium was stored at –80℃ until use. For the in vivo application, MSC-CM was collected following the above method and concentrated 50 fold by centrifugation at 5000 × *g* for 60–70 min.

### Animal treatments

Male (8 weeks old) and female (9–10 months old) C57BL/6 J mice were purchased from Charles River Laboratories. Postnatal day 4 (PD4) and 4-week-old female C57BL/6 J mice were obtained from the animal center of Shandong University. All mice were provided with a standard diet and water *ad libitum* and housed under a 12/12-h light/dark cycle.

Four-week-old female mice were injected with VCD (80 mg/kg) for 10 consecutive days to induce the POI model. The control mice were injected with normal saline with 0.1% DMSO. HA was diluted to 0.3 mg/ml in sterile PBS, and UC-MSCs were preconditioned in a 5% O_2_ incubator for 48 h prior to transplantation. After anesthesia, the ovaries were exposed and the POI mice were transplanted with 5 µl HA (0.3 mg/ml) with or without UC-MSCs at a dose of 1 × 10^5^ cells per ovary. The same number of stem cells, which were pretreated with HA (0.3 mg/ml) for 5 days and with hypoxia (5% O_2_) for 48 h, were transplanted along with HA (0.3 mg/ml) into the ovaries of the aged mice. For aged mice, 5 µl MSC-CM mixed with 0.3 mg/ml HA, MSC-CM mixed with 0.3 mg/ml HA and 1 μg/ml HGF neutralizing antibody (R&D), or 800 ng/ml HGF (Peprotech) mixed with 0.3 mg/ml HA were injected as described above. The mice were kept under observation under a warming lamp after surgery until they recovered from anesthesia.

### Estrous cycle

Estrous cycles were evaluated by vaginal smears for 14 consecutive days. After the vaginal introitus of the mouse was washed with saline four times, exfoliated epithelial cells were collected and smeared onto glass slides. The smears were air-dried, fixed in 95% ethanol, and stained with hematoxylin and eosin. Cell types and their proportions were quantified under a microscope, and estrous stages were determined according to the established criteria [22].

### Ovarian morphological analysis and follicle counting

After mice were euthanized, the ovaries were collected and fixed in Bouin’s solution overnight and then dehydrated through a graded ethanol series, vitrified in xylene, and embedded in paraffin. Next, ovaries were sliced into 5 μm thick sections, deparaffinized with xylene, and subjected to H&E staining. Slides were examined and photographed under a microscope (Olympus). Follicles were counted in one of every five sections, and only follicles with an obvious nucleus in the oocyte were counted. The total number of follicles was obtained by multiplying by 5. According to the classification method, primordial follicles were defined as oocytes surrounded by a single layer of squamous granulosa cells, primary follicles had enlarged oocytes and one layer of cuboidal granulosa cells, secondary follicles had more than one layer of cuboidal granulosa cells without an antral space, and antral follicles had antral spaces [23].

### Fertility test

Male mice at 8 weeks of age were mated with naturally aging or VCD-treated female mice at a 1:2 ratio for 4 or 6 months after transplantation. The number of offspring delivered per female was recorded three times a week, and males were randomly rotated among cages after each pregnancy.

### Serum hormone levels and enzyme-linked immunosorbent assay (ELISA)

A total of 0.8–1 ml blood was collected from the posterior orbital venous plexus of the eye after anesthesia, and the samples were incubated at 4 °C overnight. Upon coagulation, the samples were centrifuged at 2,000 rpm for 30 min, and the serum samples were sent to Beijing North Institute of Biological Technology (Beijing, People’s Republic of China) for FSH and E_2_ measurements using the radioimmunoassay method. Cytokine levels of VEGF, HGF, EGF, and SCF in MSC-CM and HA/MSC-CM were measured by the same institute using ELISA kits (Ebioscience). Prior to each assay, the conditioned media were concentrated 50-fold as described above.

### Immunohistochemistry and immunofluorescence

The slides were immersed in xylene and rehydrated through a series of alcohol gradients, and antigen retrieval was performed by boiling for 30 min in sodium citrate buffer. Endogenous peroxidase activity was quenched by incubating in 3% hydrogen peroxide for 15 min at room temperature. After blocking with 10% goat serum with 0.3% Triton X-100 (Sigma) at 37 °C for 1 h, the sections were immunolabeled with anti-DDX4 (Abcam) or anti-cKit antibody (Dako) at 4 °C overnight and subsequently incubated with goat anti-rabbit/mouse secondary antibody (ZSGB-BIO) for 1 h. Finally, the sections were stained with DAB (Vector) and counterstained with hematoxylin. Immunofluorescent staining for anti-human nucleus antibody (Chemicon) was performed in the same way as IHC on the first day but without incubating with hydrogen peroxide. On the second day, these sections were stained with secondary antibody conjugated to Alexa Fluor 488 for 1 h at room temperature. After counterstaining with DAPI, the fluorescence signal was detected under a fluorescence microscope (Olympus).

### Cytokine array

To determine the secretory profile of the culture supernatants, the 25-fold concentrated conditioned media were used with a protein array containing 440 human cytokines (RayBiotech) along with antibodies to determine the protein expression levels. Differential gene expression analysis and pathway analysis were also performed by RayBiotech (Guangzhou, China).

### Ovarian culture

Ovaries were dissected from PD4 mice and washed three times in Leibovitz’s-15 medium (Gibco) containing 10% fetal bovine serum plus 1% penicillin–streptomycin before being transferred to culture inserts (Millipore) in a 6-well culture plate (Costar) at 37℃ and 5% CO_2_. DMEM/F12 (Gibco) supplemented with 5% Insulin-Transferrin-Selenium (Sigma), 1 mg/ml BSA (Sigma), 1 mg/ml Albumax II (Gibco), 100 µM L-ascorbic (Sigma), and 1% penicillin–streptomycin was used as the culture medium. A drop of medium was placed to cover the top of the ovary to prevent drying, and the ovaries were cultured for 4 or 8 days with medium changed every 2 days. Ovaries were treated with control medium (1% DMSO), VCD (30 µM), VCD + MSC-CM (fivefold concentration), or VCD + Fib-CM (fivefold concentration). Appropriate concentrations of recombinant human HGF (100–800 ng/ml), G-CSF (100–800 ng/ml), BDNF (100–800 ng/ml), or HGF neutralizing antibody (0–1 ng/ml) were added directly to the culture medium.

### TUNEL assay

After deparaffination and rehydration, the ovarian sections were incubated with 20 µg/ml proteinase K for 15 min at room temperature, washed with PBS, and incubated with the TUNEL reagent (Roche) for 1 h at 37 °C. The nuclei were then counterstained with DAPI, and the images were captured by fluorescence microscopy (Olympus).

### Western blot

Mouse ovaries were collected, and the protein was extracted with total protein extraction kits (Invent) containing phosphatase and protease inhibitor cocktails (Sigma). A BCA Protein Assay Kit (Thermo) was used to determine the protein concentration. The protein samples were separated on 8%–12% SDS-PAGE gels and then transferred onto polyvinylidene difluoride membranes (Millipore). Membranes were blocked with 5% non-fat dry milk for 2 h and incubated overnight at 4 °C with the following primary antibodies: phospho-cKit (Try719, CST), c-Kit (CST), phospho-AKT (Ser473, CST), AKT (CST), β-actin (CST), Bad (Abcam), Bax (Abcam), cleaved PARP1 (CST), S6K1 (CST), phospho-S6K1 (Thr389, CST), phospho-rpS6 (Ser240/244, CST), rpS6 (CST), DDX4 (Abcam), SOHLH1 (Abcam), and MSY2 (Peprotech). The membranes were then incubated with horseradish peroxidase-conjugated anti-mouse or anti-rabbit secondary antibodies (Proteintech) for 1 h at room temperature. Signals were detected with enhanced chemiluminescent substrate (Millipore).

### Statistical analysis

The statistical analyses were performed with SPSS 25.0 software, and the values were expressed as the mean ± SD. Two-tailed student’s *t* test was applied for analyzing the statistical differences between two groups. The statistical differences among three or more groups were analyzed by one-way analysis of variance (ANOVA) with LSD multiple comparison analysis. The Kruskal–Wallis test was conducted as a nonparametric test when appropriate. The differences were considered statistically significant when the *P-*value was less than 0.05.

## Results

### HA prolonged UC-MSCs retention in recipient ovaries

The UC-MSCs used in this study were first characterized. The spindle-shaped UC-MSCs were arranged closely with a vortex growth pattern (Additional file 1: Figure S1A). Flow cytometry showed that the cells were positive for CD90, CD105, and CD73, but negative for CD34, CD31, CD45, and HLA-DR (Additional file 1: Figure S1B-I). In addition, the cells could be successfully induced to differentiate into osteocytes, adipocytes, and chondrocytes (Additional file 1: Figure S1J-L).

To determine whether HA could serve as a scaffold for ovarian MSCs transplantation, we first evaluated the properties of the UC-MSCs after HA pretreatment in vitro. The viability of UC-MSCs decreased significantly when cultured with 0.5 mg/ml HA for 5 days, whereas no notable differences were observed with 0.1 or 0.3 mg/ml HA (Additional file 1: Figure S2A). Furthermore, RNA-Seq was performed to determine the changes in gene expression in UC-MSCs when cultured with 0.3 mg/ml HA. Compared with the control group, among the 29,207 genes examined, the expression of 99.99% of these genes remained unchanged in the HA-treated group (Additional file 1: Figure S2B and Additional file 2: Table S1), thus indicating that HA exerts little influence on the transcriptomes of UC-MSCs. In order to track the transplanted human UC-MSCs in vivo, we selected the different sequences of human and mouse *GAPDH* genes and designed two pairs of primers, which were specific for human *GAPDH* gene (F: 5'-GCACCCTATGGACACGCTC-3', R: 5'-CCCACATCACCCCTCTACCTC-3') and mouse *GAPDH* gene (5'-GTCACTACCGAAGAACAACGAG-3', R: 5'-TGTGGGCTCCGAACTGAT-3'), respectively. After transplantation of UC-MSCs combined with different concentrations of HA (0, 0.1, 0.2, 0.3 mg/ml) into mouse ovaries, we found that 0.3 mg/ml HA promoted stem cell retention to the greatest extent at the day after transplantation (Additional file 1: Figure S2C). Therefore, the concentration of 0.3 mg/ml HA was used in the subsequent experiments.

**Fig. 1:**
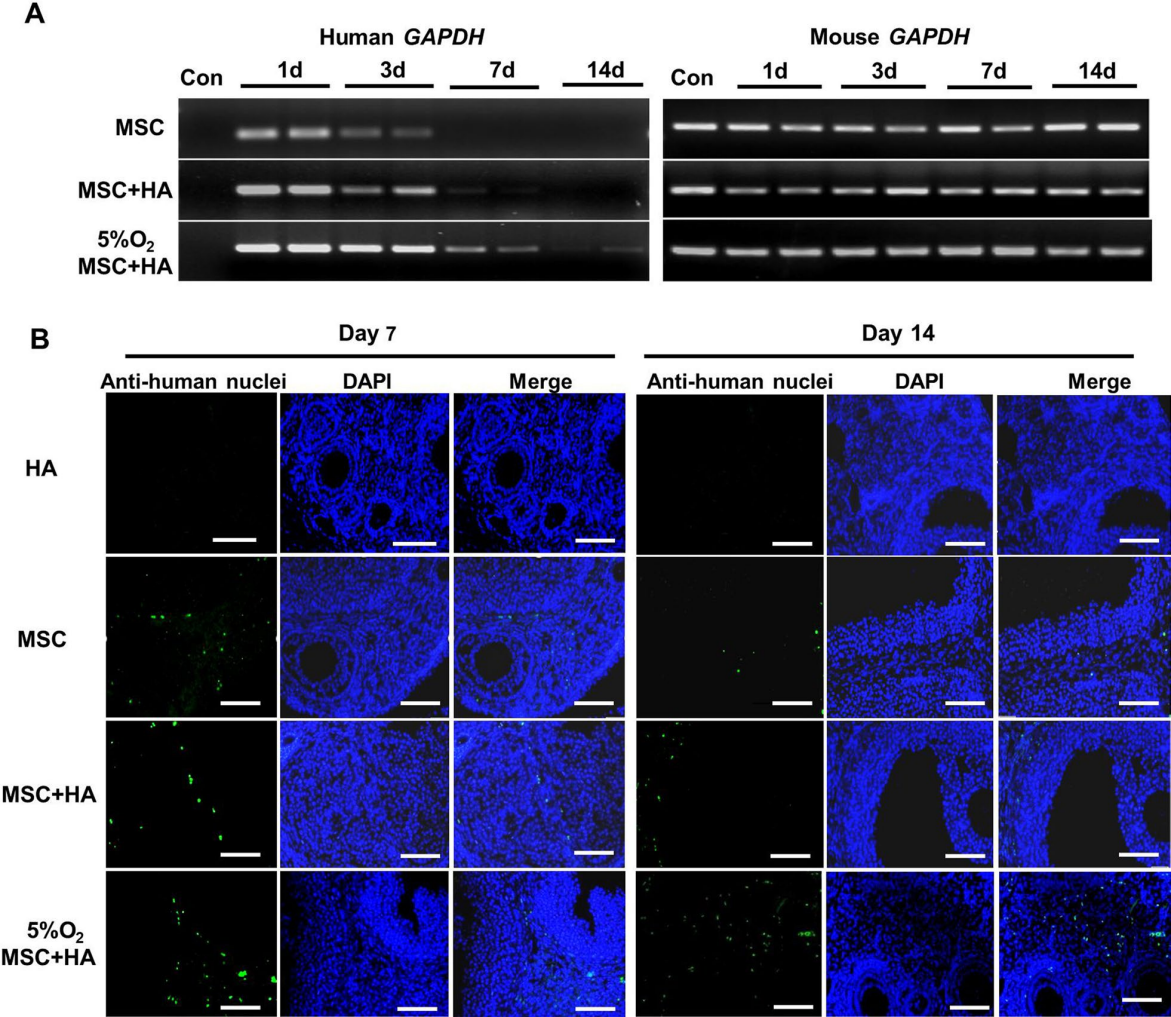
HA prolonged UC-MSCs retention in recipient ovaries. **a** The stem cell tracing by agarose gel electrophoresis for PCR amplification of human and mouse *GAPDH* genes in the MSC group, MSC + HA group, and 5% O_2_ MSC + HA group at different times after transplantation. **b** At 1 or 2 weeks after transplantation, the distribution of UC-MSCs in the three groups was detected by immunofluorescence staining against human nuclei (green). UC-MSCs were mainly located in the ovarian stroma tissue. The nuclei were counterstained with DAPI (blue). Scale bars = 50 μm

To further evaluate the impact of HA on the residence time of transplanted UC-MSCs in the ovaries, we tracked them in vivo for 14 days using PCR assays with specific primers for the human *GAPDH* gene. In the HA supplementation group, PCR products could be observed until 7 days after UC-MSCs transplantation, whereas in the non-HA group the positive bands were no longer detected after 3 days, thus indicating that the retention time of UC-MSCs combined with HA was significantly prolonged (Fig. 1a). Considering the favorable effects of hypoxia on stem cell survival [24], hypoxic priming was also applied in this study and the positive bands could be detected after 14 days (Fig. 1a). In addition, immunofluorescence staining also confirmed that the combination strategy with hypoxia preconditioning and HA improved UC-MSCs survival in the stroma at 14 days after transplantation (Fig. 1b). Collectively, these results indicated that UC-MSCs combined with HA after hypoxia pretreatment was an effective method for MSC transplantation in the ovary.

### UC-MSCs combined with HA efficiently and safely rescued the loss of follicles in VCD-induced POI mice

To investigate the therapeutic effect of UC-MSCs combined with HA (MSC/HA) on ovarian function, a VCD-induced POI mouse model was established. VCD is an ovotoxic chemical that causes apoptosis of primordial and primary follicles [25]. The workflow for evaluating stem cell therapy is shown in Additional file 1: Figure S3. The mice were randomly divided into the following four groups: 1) control group, 2) VCD group, 3) VCD + HA transplantation group, and 4) VCD + MSC/HA transplantation group.

**Fig. 2:**
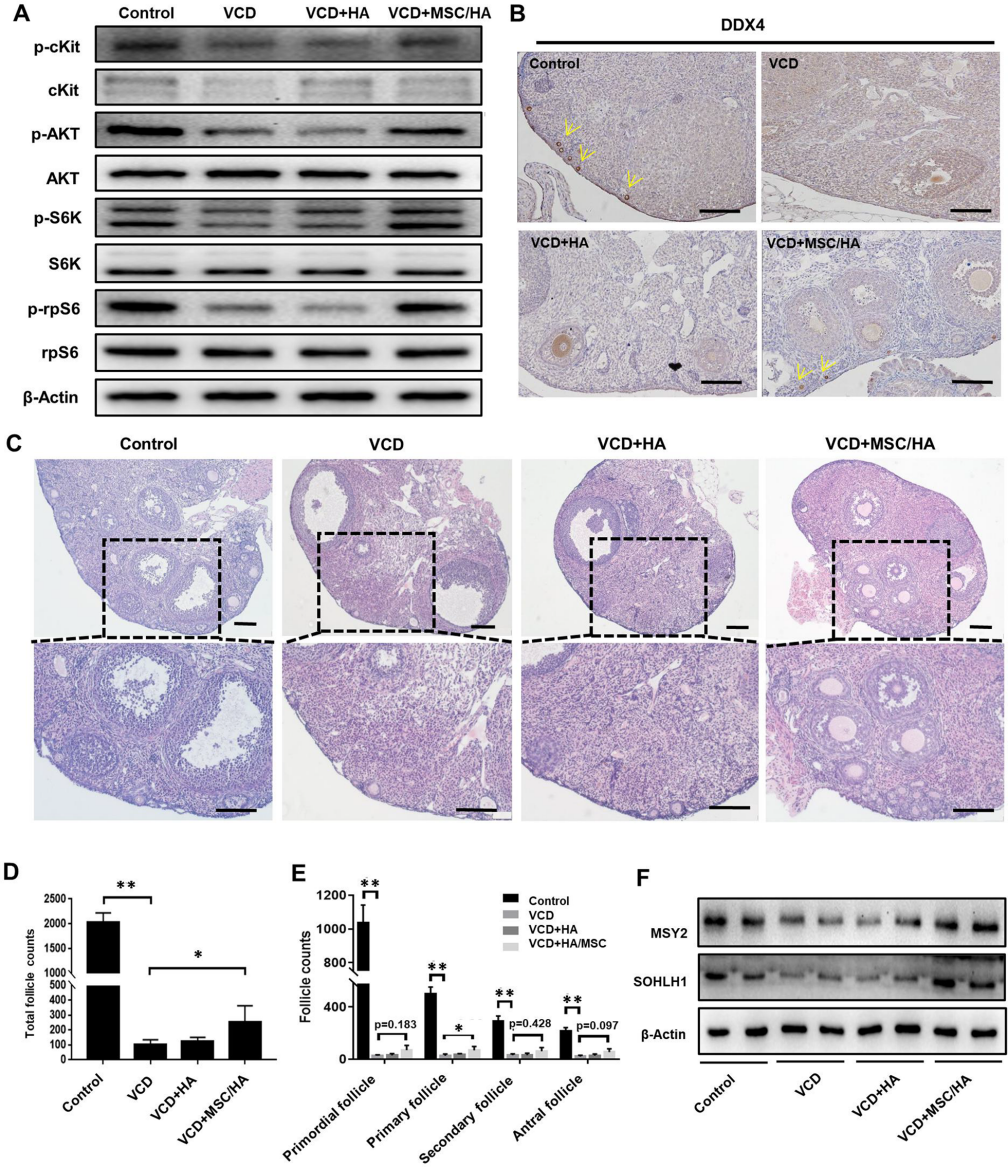
MSC/HA transplantation activated the PI3K-AKT pathway and increased follicle numbers in POI mouse model. **a** Western blot showing the activation of the PI3K-AKT pathway in the control, VCD, VCD + HA, and VCD + MSC/HA groups 4 days after transplantation. **b** Immunostaining showing the expression of DDX4 in ovaries 1 week after transplantation in the four groups. The yellow arrows indicate primordial follicles. Scale bars = 50 μm. **c** H&E staining of ovaries from the four groups at 10 weeks after transplantation. Scale bars = 100 μm. **d** Numbers of total follicles and **e** follicles at various stages were counted at 10 weeks after transplantation and compared with VCD group (n = 8 for each group). *P < 0.05, **P < 0.01. **f** Western blot showing the expression of germ cell markers MSY2 and SOHLH1 in ovaries from the four groups at 10 weeks after transplantation

VCD was injected intraperitoneally into mice for 10 days, and then MSC/HA or HA were transplanted into the ovaries. After 4 days, the activation of the PI3K-AKT pathway was measured. Decreased expression of cKit, p-cKit, p-AKT, p-S6K, and p-rpS6 was observed in the VCD group, while restored levels of these proteins were found in the MSC/HA group, and HA alone did not exert similar effects (Fig. 2a). Furthermore, the apoptosis-associated proteins, such as cleaved PARP1, BAX, and BAD, were significantly up-regulated in the VCD-induced POI mice compared with controls, and as expected MSC/HA injection partially inhibited apoptosis (Additional file 1: Figure S4A). Additionally, H&E staining and western blot showed more follicles and germ cells at one week after MSC/HA transplantation compared with the VCD and HA groups (Additional file 1: Figure S4B and C), and this was confirmed by immunostaining with the germ cell marker DDX4 (Fig. 2b).

To explore the effects of MSC/HA on the resumption of ovarian function, we measured follicle numbers and sex hormones, including follicle-stimulating hormone (FSH) and estradiol (E_2_), at 10 weeks after transplantation. The morphological differences between the ovaries of the four groups were obvious (Fig. 2c). The total follicle number showed a significant reduction in the VCD group (*P* < 0.01), which was increased after MSC/HA transplantation (*P* = 0.041) (Fig. 2d). In addition, the follicles at different stages had an upward tendency in the MSC/HA group (primordial follicle, *P* = 0.183; primary follicle, *P* = 0.03; secondary follicle, *P* = 0.428; secondary follicle, *P* = 0.097) (Fig. 2e). Consistent with this, the expression levels of the germ cell markers SOHLH1 and MSY2 were also elevated after MSC/HA transplantation (Fig. 2f). However, the mice in the four groups showed regular estrous cycles and comparable levels of FSH and E_2_ (Additional file 1: Figure S5A and B).

To further assess the restoration of ovarian function after MSC/HA transplantation, fertility tests were performed for 6 months (Additional file 2: Table S2). The VCD + MSC/HA group showed a tendency for a higher average number of pups/litter and litters/mouse compared with the VCD group (pups/litter 6 ± 1.91 vs. 4.18 ± 1.99; litters/mouse 2.25 ± 1.16 vs 1.57 ± 0.98), although the differences did not reach statistical significance. For safety evaluation, no tumorigenesis was observed except for hematoma in two ovaries from the VCD + MSC/HA group (Additional file 1: Figure S6 A and B). In addition, there were no obvious abnormalities in the offspring from any group. In summary, MSC/HA transplantation could prevent VCD-induced follicle loss efficiently and safely in mice.

### UC-MSCs facilitated follicle survival through a paracrine mechanism both in vitro and in vivo

**Fig. 3:**
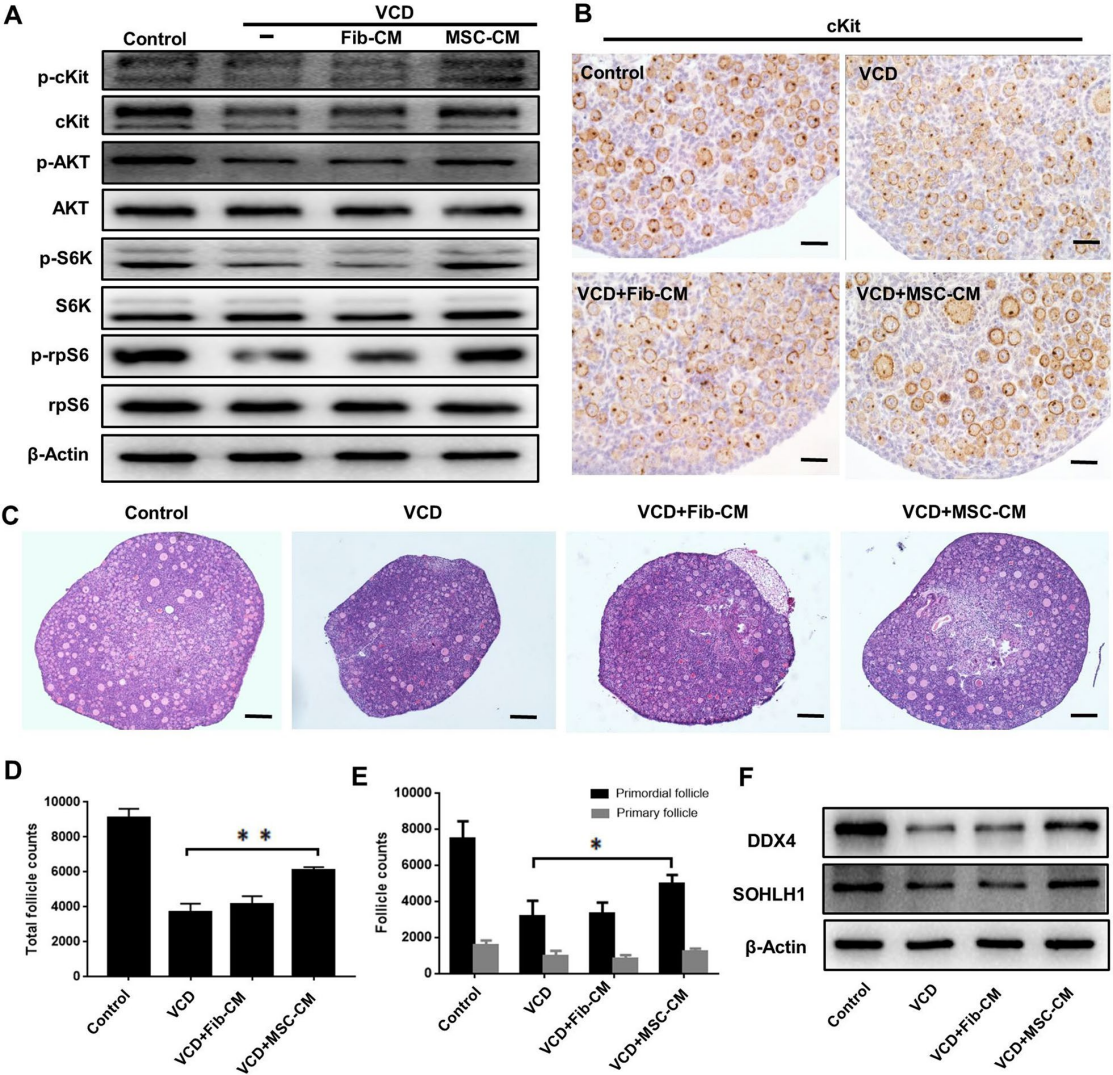
UC-MSCs facilitated follicle survival in a paracrine manner. **a** The activation of the PI3K-AKT pathway was measured in cultured ovaries from the control, VCD, VCD + Fib-CM, and VCD + MSC-CM groups by western blot. **b** Immunostaining showed the expression of cKit in ovaries from different groups after 4 days of culture. Scale bars = 50 μm. **c** H&E staining showing the ovarian morphology after 8 days of in vitro culture in the four groups. Scale bars = 100 μm. **d** The numbers of total follicles and **e** follicles at various stages were counted and compared after culturing for 8 days (n = 3 for each group) *P < 0.05, **P < 0.01. **f** Western blot showing the DDX4 and SOHLH1 expression after 8 days of ovarian culture in the four groups

Accumulating evidence suggests that paracrine effects may be more instrumental in stem cell therapy than the effects of cell differentiation [26]. Therefore, we further explored whether UC-MSCs ameliorated ovarian damage via a paracrine mechanism in vitro. We cultured PD4 mouse ovaries that were treated with VCD in the presence of concentrated UC-MSCs and fibroblast-conditioned media (MSC-CM or Fib-CM). After 4 days, the expression levels of cKit, p-cKit, p-AKT, p-S6K, and p-rpS6 in the VCD group were down-regulated, whereas increased expression of these proteins was observed in the VCD + MSC-CM group, but no changes were seen in the VCD + Fib-CM group (Fig. 3a). Consistent with this, immunohistochemistry showed that the reduced level of cKit induced by VCD was rescued by MSC-CM but not by Fib-CM (Fig. 3b). In addition, MSC-CM effectively mitigated the cell apoptosis induced by VCD in vitro (Additional file 1: Figure S7 A and B).

We further performed H&E staining of ovaries cultured in vitro. The ovaries in the VCD and VCD + Fib-CM groups were smaller, whereas ovaries in the VCD + MSC-CM group had a similar size as the control group (Fig. 3c). In addition, follicle counting showed that the numbers of total follicles (*P* = 0.004) and primordial follicles (*P* = 0.011) were significantly increased in the VCD + MSC-CM group compared with the VCD group (Fig. 3d and e). Consistent with this, the expression levels of DDX4 and SOHLH1 were both elevated in the VCD + MSC-CM group (Fig. 3f).

To determine whether the therapeutic effect of MSCs is via a paracrine mechanism in vivo, MSC-CM combined with HA (MSC-CM/HA) was injected into the ovaries of VCD-induced POI mice. At 7 days after transplantation, MSC-CM/HA partially alleviated the reduction in DDX4 (Additional file 1: Figure S8), indicating that more germ cells had survived. Taken together, these results illustrated that UC-MSCs could facilitate follicle survival via a paracrine mechanism.

### HGF secreted by UC-MSCs promoted follicle survival via activation of the PI3K-AKT pathway

**Fig. 4:**
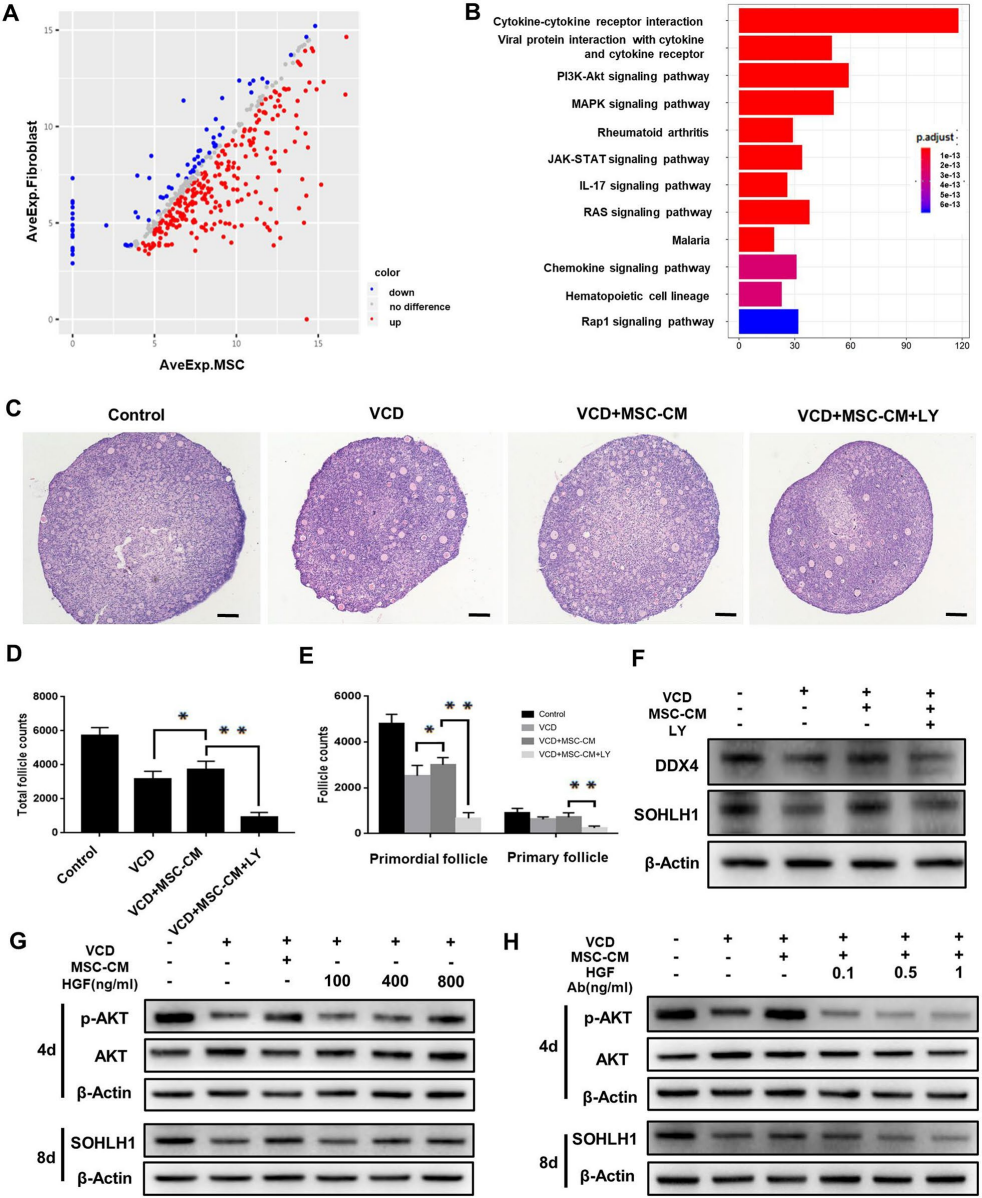
HGF released from UC-MSCs promoted follicle survival via activation of the PI3K-AKT signaling pathway. **a** Scatter diagram showing the differentially expressed proteins between MSC-CM and Fib-CM using the 440-cytokine array. **b** KEGG pathway analysis exhibited the enriched pathways with many genes involved in the PI3K-AKT pathway. **c** H&E staining showing the ovary morphology at 8 days after exposure to LY294002. Scale bars = 50 μm. **d** Total follicles and **e** follicles at different stages were counted in different groups and compared after culturing for 8 days (n = 6 for each group). *P < 0.05, **P < 0.01. **f** Western blot showing the expression of germ cell markers SOHLH1 and DDX4 in the four groups after culturing for 8 days. **g** The expression of p-AKT and SOHLH1 in VCD-impaired ovaries after the addition of different concentrations of HGF was detected by western blot after 4 and 8 days of in vitro culture, respectively. **h** Western blot showing the expression of p-AKT and SOHLH1 in VCD-impaired ovaries after in vitro culturing for 4 and 8 days, respectively, in MSC-CM supplemented with different concentrations of HGF neutralization antibody (HGF Ab)

To identify the effective components secreted by UC-MSCs, MSC-CM and Fib-CM were subjected to a cytokine array. Cytokines in MSC-CM with fold change > 1.2 or < 0.83 compared with Fib-CM were analyzed. A total of 328 differentially expressed cytokines were identified, including 255 genes that were up-regulated in MSC-CM (Fig. 4a and Additional file 2: Table S3). Moreover, Kyoto Encyclopedia of Genes and Genomes (KEGG) enrichment revealed that 59 differential cytokines were related with the PI3K-AKT pathway, which ranked first among all pathways that were analyzed (Fig. 4b and Additional file 2: Table S4).

To evaluate the role of the PI3K-AKT pathway during the repair process, LY294002, a highly selective PI3K inhibitor, was added to MSC-CM in cultured ovaries. The effect of MSC-CM in preventing follicle loss was abrogated when LY294002 was added (Fig. 4c), and substantial loss of follicles was also observed when blocking this pathway (Fig. 4d and e). The decreased expression of DDX4 and SOHLH1 further indicated reduced germ cell survival when the PI3K pathway was blocked (Fig. 4f). Together, these results suggested that MSC-CM exerted its protective effect on follicle survival via the PI3K-AKT pathway.

To determine which components play a key role in follicle survival, we further explored the effective components that activate the PI3K-AKT pathway. The standards were set up as follows: (1) chemiluminescence signal > 150; (2) fold change > 2 (Additional file 2: Table S5). According to the fold change, the four cytokines including HGF, PDGF-BB, G-CSF, and BDNF, with fold changes > 20 among the 22 tested cytokines, were selected. Given that PDGF-BB has little effect on follicle protection [27], we focused on HGF, G-CSF, and BDNF in the subsequent experiments. The effects of HGF, G-CSF, and BDNF on VCD-induced ovary damage were evaluated in vitro. Intriguingly, HGF supplementation elevated SOHLH1 expression in a dose-dependent manner (Fig. 4g), but G-CSF and BDNF did not exert similar effects (Additional file 1: Figure S9A and B). It was identified that HGF at a concentration of 800 ng/ml could effectively activate the PI3K-AKT pathway after 4 days of ovary culture (Fig. 4g). To further confirm the effect of HGF, a neutralizing antibody against HGF was added to the MSC-CM. The PI3K-AKT activation was attenuated, and the follicle protection of MSC-CM was also weakened in a dose-dependent manner when HGF was neutralized (Fig. 4h). Together, HGF was identified as the key component to exert the therapeutic effect of MSC-CM via activating the PI3K-AKT pathway.

### HA reinforced the paracrine ability of UC-MSCs to activate the PI3K-AKT pathway

**Fig. 5:**
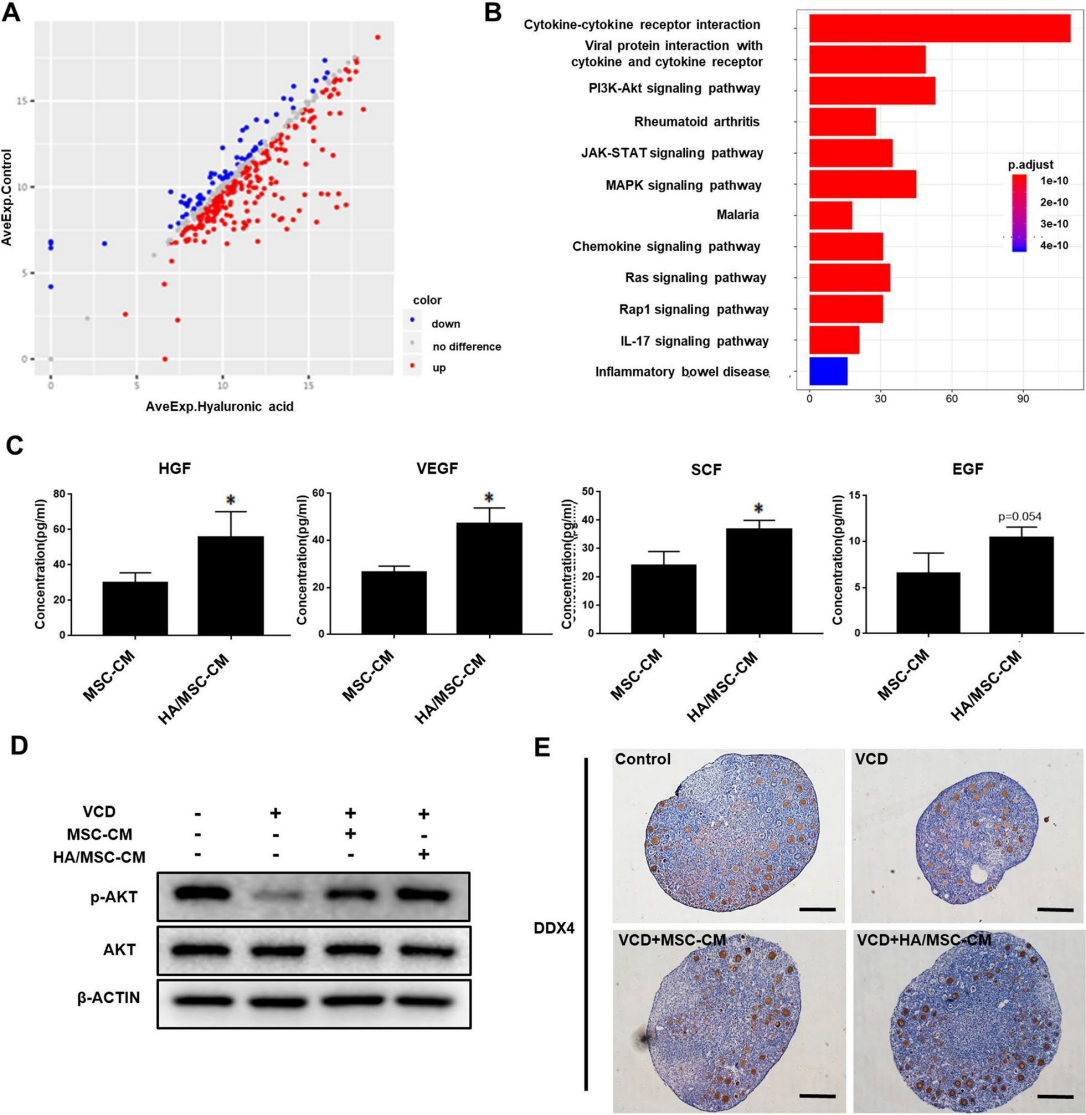
HA reinforced the paracrine ability of UC-MSCs to further activate the PI3K-AKT pathway and promote follicle survival. **a** Scatter diagram showing the differentially expressed proteins between MSC-CM and HA/MSC-CM according to the 440-cytokine array. **b** KEGG pathway analysis showing many differentially expressed proteins related to the PI3K-AKT signaling pathway. **c** The levels of HGF, VEGF, SCF, and EGF were measured by ELISA in MSC-CM and HA/MSC-CM (n = 3). *P < 0.05. **d** Western blot showing the activation of the PI3K-AKT pathway by MSC-CM and HA/MSC-CM. **e** Immunostaining showing the expressions of DDX4 in the ovaries in the four groups after culturing for 8 days. Scale bars = 50 μm

Next, to determine the effect of HA on the paracrine function of stem cells, MSC-CM with or without 5 days of HA pretreatment (HA/MSC-CM) was collected and subjected to cytokine array analysis. The results showed that the secretion of many cytokines was obviously up-regulated after HA treatment (Fig. 5a and Additional file 2: Table S6). Interestingly, many differentially expressed cytokines were related to the PI3K-AKT pathway according to KEGG pathway analysis (Fig. 5b and Additional file 2: Table S7). These results suggest that HA can potentiate UC-MSCs’ paracrine function.

To further confirm the effect of HA, follicle protective cytokines in MSC-CM with or without HA treatment were analyzed by ELISA assay. HGF as well as other cytokines such as vascular endothelial growth factor (VEGF) and stem cell factor (SCF) were up-regulated by HA treatment (*P* = 0.046, 0.007, and 0.02, respectively, Fig. 5c). Epidermal growth factor (EGF) also showed an increasing tendency but did not reach statistical significance (*P* = 0.054, Fig. 5c). Interestingly, higher expression of p-AKT was observed in ovaries treated with VCD + HA/MSC-CM compared to the VCD + MSC-CM group, indicating enhanced activation of the PI3K-AKT pathway (Fig. 5d). In addition, immunostaining of DDX4 suggested that more germ cells were rescued with HA/MSC-CM than MSC-CM after 8 days of culture (Fig. 5e). This suggests that HA reinforced the paracrine function of UC-MSCs in activating the PI3K-AKT pathway to promote follicle survival.

### MSC/HA transplantation mitigated age-related fertility impairment in mice

**Fig. 6:**
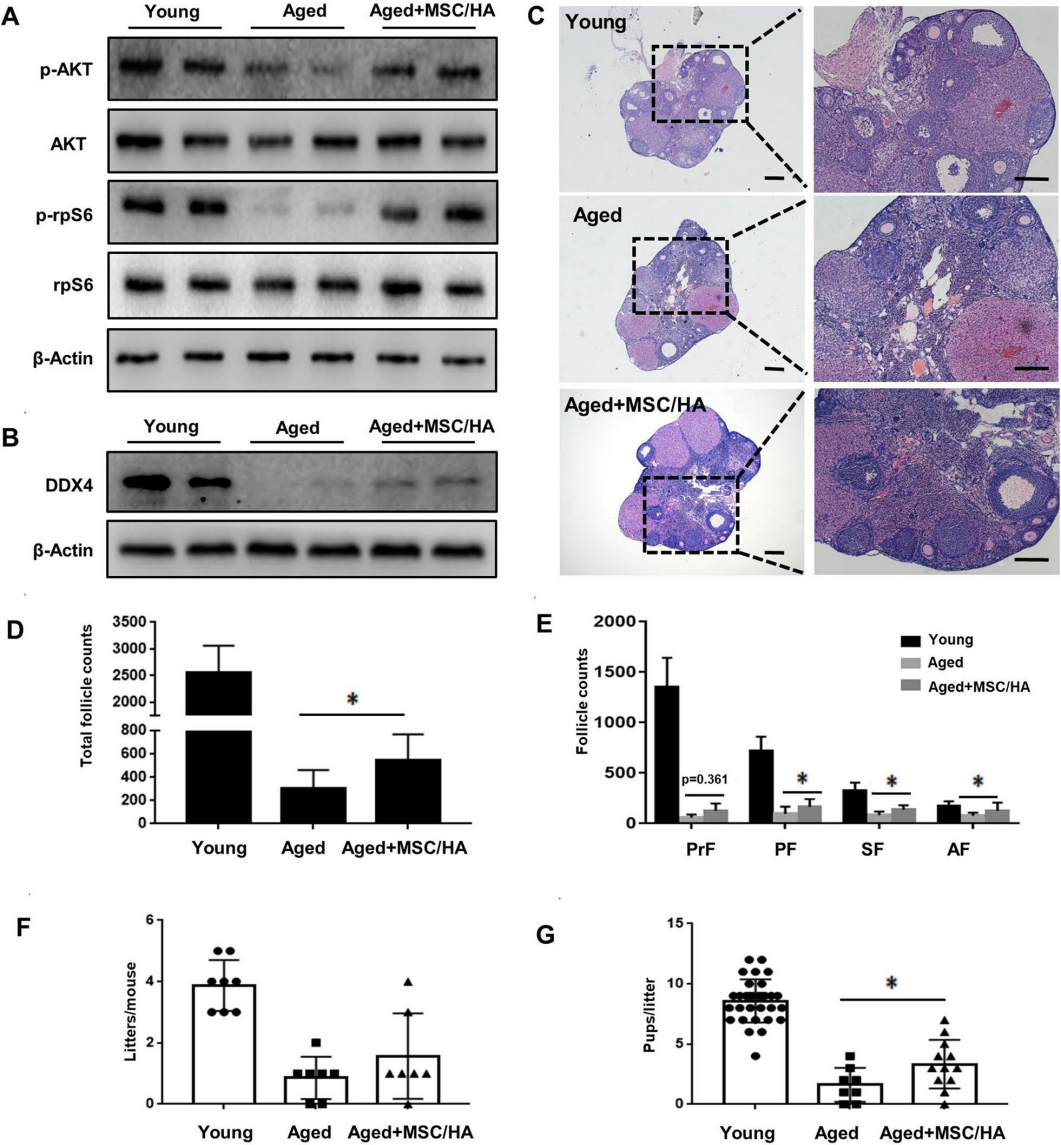
MSC/HA transplantation mitigated age-related fertility impairment in mice. **a** Western blot showing the expression of p-AKT and p-rpS6 in the ovaries of young, aged, and aged + MSC/HA mice 4 days after transplantation. **b** Western blot and **c** H&E staining showing germ cell marker DDX4 expression and follicle preservation at 8 weeks after transplantation in the three groups. Scale bars = 200 μm. **d** The numbers of total follicles and **e** follicles at various stages were counted and compared in the three groups 8 weeks after transplantation (n = 5 for each group). *P < 0.05. PrF, primordial follicle; PF, primary follicle; SF, secondary follicle; AF, antral follicle. **f**, **g** Comparison of the average number of litters and number of pups per litter between aged mice and MSC/HA-treated aged mice (n = 7 or 8 for each group). *P < 0.05

Finally, we used 10-month-old mice to assess the potential therapeutic effect of MSC/HA transplantation on natural ovarian aging. Four days after transplantation, elevated levels of p-AKT and p-rpS6 suggested activation of the PI3K-AKT pathway (Fig. 6a). Eight weeks later, increased DDX4 expression indicated that more germ cells survived (Fig. 6b). Compared with young mice (4 months old), the ovaries of aged mice had fewer follicles. In contrast, after MSC/HA injection more follicles in aged ovaries were preserved (Fig. 6c and d). As expected, follicles at different stages showed increasing tendencies after MSC/HA transplantation (primordial follicles, *P* = 0.361; primary follicles, *P* = 0.046; secondary follicles, *P* = 0.003; antral follicles, *P* = 0.032) (Fig. 6e). Fertility testing was also carried out for 4 months after stem cell transplantation. Aged mice showed obviously decreased fertility compared with young mice (Additional file 2: Table S8) (pups/litter, 1.63 ± 1.41 vs. 8.58 ± 1.79, *P* < 0.01; litters/mouse, 1.57 ± 1.40 vs. 3.88 ± 0.84, *P* < 0.01), whereas the average number of pups/litter in aged mice after MSC/HA treatment was about twofold that of those without MSC/HA transplantation (3.33 ± 2.02 vs. 1.63 ± 1.41; *P* = 0.042, Fig. 6g). However, the increase in litters/mouse did not reach statistical significance (1.57 ± 1.40 vs. 0.86 ± 0.69, *P* = 0.202, Fig. 6f). In addition, after 4 months of fertility testing no tumorigenesis was observed in any of the ovaries (Additional file 1: Figure S10), and no obvious abnormalities were seen in any of the offspring. In summary, UC-MSCs exhibited effective and safe therapeutic effects on natural aging ovaries in mice.

Furthermore, to confirm the paracrine action and the role of HGF in the resumption of ovarian function in aged mice, MSC-CM, MSC-CM mixed with HGF antibody, and HGF alone were injected into mouse ovaries. Four days later, the level of p-AKT was elevated after MSC-CM and HGF treatment (Additional file 1: Figure S11A). Similarly, 8 weeks later the ovaries treated with MSC-CM and HGF showed higher DDX4 expression (Additional file 1: Figure S11B). In contrast, the effects of MSC-CM were dramatically reduced after the addition of HGF antibody (Additional file 1: Figure S11A and B). These in vivo results further suggest the paracrine action of UC-MSCs and the key role of HGF in follicle protection.

## Discussion

In this study, we used the novel combination of UC-MSCs with autocrosslinked HA for transplantation to prevent both physiological and pathological ovarian aging in mice. As a scaffold with great prospects for clinical application, HA not only prolonged UC-MSCs retention in the ovary, but also potentiated its secretory function. Mechanistically, UC-MSC promoted follicle survival by activating the PI3K-AKT pathway through a paracrine mechanism both in vitro and in vivo, and HGF was identified as the key functional component in the UC-MSCs secretome.

Although stem cell therapy has shown great promise in both basic and some clinical studies, the cells’ limited survival and poor efficiency greatly attenuate their clinical application [7, 28]. Different scaffolds and hydrogels have been applied to try to improve MSC retention in animals, and fibrous scaffolds and membrane-binding adhesive particulates have been constructed to augment MSCs’ paracrine ability [29, 30]. As a linear polysaccharide existing in the extracellular matrix (ECM) and constituting the microenvironment of MSCs in the physiological state, HA has been considered as a promising material to foster tissue regeneration [11, 31]. Engineered HA-based biomaterials have been used to mimic the ECM, and HA has been shown to potentiate proliferation, delay senescence, and maintain the stemness of MSCs [32, 33]. It is noteworthy that the autocrosslinked HA applied in this study has been approved by the China Food & Drug Administration for the prevention of intrauterine adhesions in the clinic. In this work, we combined UC-MSCs with autocrosslinked HA as a scaffold for transplantation into the ovaries. Intriguingly, HA not only contributed to longer retention time, but also strengthened the paracrine function of UC-MSCs, as indicated by the increased secretion of cytokines such as HGF, VEGF, SCF, and EGF. Although it is promising to use HA for priming MSCs before transplantation, the exact mechanisms behind these effects await further exploration. In addition, it has been reported that age-associated ovarian stiffness is dependent on increased collagen deposits and decreased HA content [34], which supports the feasibility of using HA as a scaffold for transplantation of stem cells into the ovary. Considering that VEGF supports ovarian angiogenesis [35], SCF is responsible for both primordial follicle survival and activation [36, 37], and EGF improves primordial follicle activation [38], these cytokines might also contribute to follicle growth and development after stem cell transplantation in the ovary. In all, HA is considered to be a feasible scaffold for clinical stem cell transplantation in patients with ovarian disorders.

At present, despite the plausibility that MSCs can rescue ovarian function by enhancing cell proliferation, preventing oxidation and fibrosis, and modulating the immune system [39], little is known about the signaling pathways that are involved. It is well known that the PI3K-AKT pathway is a primary pathway for maintaining primordial follicle survival and for regulating primordial follicle activation, determining female reproductive lifespan [40]. In vitro activation technology has been used to treat infertility patients by treating ovarian tissue with PTEN inhibitor and PI3K activator, which suggests the feasibility of improving ovarian function by activating the PI3K-AKT pathway [41]. On the other hand, previous studies have shown that S6 protein kinase (S6K1), the downstream effector of this pathway, phosphorylates 40S ribosomal protein S6 (rpS6) and thus regulates protein translation and ribosome biogenesis in order to maintain the primordial follicle pool [16]. When this pathway is suppressed, the survival of primordial follicles is compromised [17]. It has been reported that brown adipose tissue xenotransplant can prolong the ovarian lifespan by improving follicle survival via activation of the PI3K-AKT pathway [42]. In this study, VCD-induced POI mice and natural aging mice both exhibited down-regulation of the PI3K-AKT pathway in the ovary, and UC-MSCs as well as MSC-CM transplantation could activate the pathway, promote the survival of primordial follicles, and ultimately improve fecundity. Moreover, in vitro experiments showed that PI3K inhibition completely blocked the effects of MSC-CM on cultured ovaries. Collectively, our results indicated that UC-MSCs as well as MSC-CM could rescue follicle loss by activating the PI3K-AKT pathway.

It has been suggested that the effectiveness of MSCs in ameliorating ovarian function is mainly attributable to paracrine mechanisms [43]. Stem cells secrete cytokines, microRNAs, extracellular vesicles, and other substances to regulate the target cells [44]. Thus, the in-depth exploration of paracrine function and the identification of effective factors will provide novel stem cell by-products suitable for clinical use. In this study, we elucidated the paracrine mechanism in vitro by ovary culture and found that MSC-CM could reverse the decreased activity of the PI3K-AKT pathway and the subsequent follicle loss induced by VCD. Consistent with this, MSC-CM could also increase the follicle survival in vivo in both POI and naturally aging mice. We further determined the functional components by cytokine array analysis and found that more cytokines were secreted by UC-MSCs compared to fibroblasts and, of particular interest, HGF presented the highest fold change. HGF was first identified as a potent mitogen for mature hepatocytes, and it has been used since then to treat degenerative disorders and acute injury [45, 46]. By using in vitro cultured ovaries and naturally aged mice, we demonstrated that exogenously supplied HGF acted as the key effector to activate the PI3K-AKT pathway and promote follicle survival. The biological effects of MSC-CM are dependent on the presence of HGF, and the protective capability was dramatically abrogated when HGF antibody was added both in vivo and in vitro. Interestingly, it was previously shown that HGF released from MSCs improved functional recovery in Alzheimer’s disease and multiple sclerosis models [47, 48]. However, the interactions between stem cells’ secretomes and ovarian target cells are not clear. It is speculated that HGF may increase the expression of SCF in granulosa cells in order to further bind with cKit on oocytes and then activate the PI3K-AKT pathway [49, 50], and in support of this, SCF has been reported to attenuate VCD-induced ovotoxicity [27]. Therefore, HGF may be considered an effective therapeutic agent to resist natural ovarian aging and improve female reproductive health.

It is generally accepted that the number of oocytes in the mammalian ovary is fixed at birth and cannot renew during the female lifespan [51], and the natural aging process poses an unfavorable threat to the survival of follicles. Although it has been reported that MSCs and MSCs-derived exosomes could improve the function of aging ovaries [52–54], the role and mechanism of stem cells in restoring ovarian function needs to be further delineated. In this study, we observed increased numbers of follicles and improved fecundity in a physiological aging mice model after stem cell therapy, further verifying its effects on follicle protection. It should be noted that 8 weeks after MSC/HA transplantation the growing follicles at the time of infusion were already exhausted, and all of the germ cells were derived from the resting primordial follicles at the time of MSC injection. Therefore, the increased numbers of follicles and germ cells should be indicative of more primordial follicles surviving after stem cell therapy. Additionally, the biosafety in the recipients’ ovaries and their progeny after MSC/HA transplantation was confirmed. Our findings thus demonstrate the effectiveness of MSC/HA in natural ovarian aging as indicated by follicle protection and rescued fecundity.

## Conclusion

In summary, we demonstrated that the autocrosslinked HA not only prolonged the local retention of UC-MSCs in the ovary, but also potentiated the paracrine function, thus showing HA’s promise in biomaterial-based stem cell therapy. We further determined the therapeutic effects of UC-MSCs/HA transplantation in both VCD-induced POI mouse model and in naturally aging mice by activating the PI3K-AKT pathway through a paracrine mechanism. In addition, HGF was identified as a critical component of MSCs in supporting follicle survival, and HGF may serve as an effective byproduct of MSCs for treating human reproductive disorders such as POI and ovarian aging.

## Supplementary Information

**Additional file 1: Figure S1.** Characteristics and differentiation potential of human UC-MSCs. **Figure S2.** Properties of UC-MSCs combined with HA *in vitro* and *in vivo*. **Figure S3.** A diagram illustrating the four groups and the chronological order exploring the effects and mechanisms of MSC/HA transplantation in VCD-induced POI mice. **Figure S4.** MSC/HA transplantation protected against VCD-induced follicle loss and alleviated cell apoptosis *in vivo*. **Figure S5.** Estrous cycles and hormone profiles in VCD-induced POI mice after transplantation. **Figure S6.** Safety assessment in the ovaries after fertility test. **Figure S7.** MSC-CM alleviated ovarian cell apoptosis induced by VCD. **Figure S8.** MSC-CM/HA was injected into the ovaries of VCD-induced POI mice, and 1 week after transplantation western blot showed the expression of DDX4 in the ovaries of the control, VCD, and MSC-CM/HA groups. **Figure S9.** BDNF and G-CSF were added to the *in vitro* ovarian culture system to determine their protective effects against VCD-induced damage. **Figure S10.** Safety assessment in the ovaries of aged mice after stem cell transplantation. **Figure S11.** HGF mediated the function of UC-MSCs in aged mice. MSC-CM, MSC-CM with HGF antibody, and HGF were injected into the aged mouse ovaries, and the ovaries in each group were collected 4 days or 8 weeks after injection and subjected to western blot analysis.

**Additional file 2: Table S1.** Differential expression genes between control and HA-pretreated UC-MSCs. **Table S2.** Fertility test results of VCD induced-POI mice after MSC combined with HA transplantation. **Table S3.** Differential expression cytokines between MSC-CM and Fib-CM. **Table S4.** KEGG pathways analysis of differential cytokines between MSC-CM and Fib-CM. **Table S5.** Twenty two up-regulated cytokines in MSC-CM related with the PI3K-AKT pathway. **Table S6.** Differential expression cytokines between MSC-CM and MSC-CM after HA pretreated. **Table S7.** KEGG pathways analysis of differential cytokines between MSC-CM and MSC-CM after HA pretreated. **Table S8.** Fertility test results of aged mice after MSC combined with HA transplantation.

## Data Availability

All data generated or analyzed during this study are included in this published article and its Additional files 1 and 2.

## Abbreviations

BDNF: Brain-derived neurotrophic factor;
E_2_: Estradiol
ECM: Extracellular matrix
EGF: Epidermal growth factor
Fib-CM: Fibroblast conditioned medium
FSH: Follicle-stimulating hormone
G-CSF: Granulocyte-colony stimulating factor
HA: Hyaluronic acid
HGF: Hepatocyte growth factor
hUC-MSCs: Human umbilical cord mesenchymal stem cells
MSC-CM: Mesenchymal stem cells conditioned medium
POI: Premature ovarian insufficiency
VEGF: Vascular endothelial growth factor
VCD: 4-vinylcyclohexene diepoxide
SCF: Stem cell factor
