# Supplementary material for: Mesenchymal stem cells combined with autocrosslinked hyaluronic acid improve mouse ovarian function by activating the PI3K-AKT pathway in a paracrine manner
Source: Stem Cell Res Ther. 2022 Feb 2;13:49. doi: 10.1186/s13287-022-02724-3 (PMC8812195; doi:10.1186/s13287-022-02724-3)
Supplement: Supplementary file 1 — Additional file 1: Figure S1. Characteristics and differentiation potential of human UC-MSCs. Figure S2. Properties of UC-MSCs combined with HA in vitro and in vivo. Figure S3. A diagram illustrating the four groups and the chronological order exploring the effects and mechanisms of MSC/HA transplantation in VCD-induced POI mice. Figure S4. MSC/HA transplantation protected against VCD-induced follicle loss and alleviated cell apoptosis in vivo. Figure S5. Estrous cycles and hormone profiles in VCD-induced POI mice after transplantation. Figure S6. Safety assessment in the ovaries after fertility test. Figure S7. MSC-CM alleviated ovarian cell apoptosis induced by VCD. Figure S8. MSC-CM/HA was injected into the ovaries of VCD-induced POI mice, and 1 week after transplantation western blot showed the expression of DDX4 in the ovaries of the control, VCD, and MSC-CM/HA groups. Figure S9. BDNF and G-CSF were added to the in vitro ovarian culture system to determine their protective effects against VCD-induced damage. Figure S10. Safety assessment in the ovaries of aged mice after stem cell transplantation. Figure S11. HGF mediated the function of UC-MSCs in aged mice. MSC-CM, MSC-CM with HGF antibody, and HGF were injected into the aged mouse ovaries, and the ovaries in each group were collected 4 days or 8 weeks after injection and subjected to western blot analysis. [file 13287_2022_2724_MOESM1_ESM.pdf]

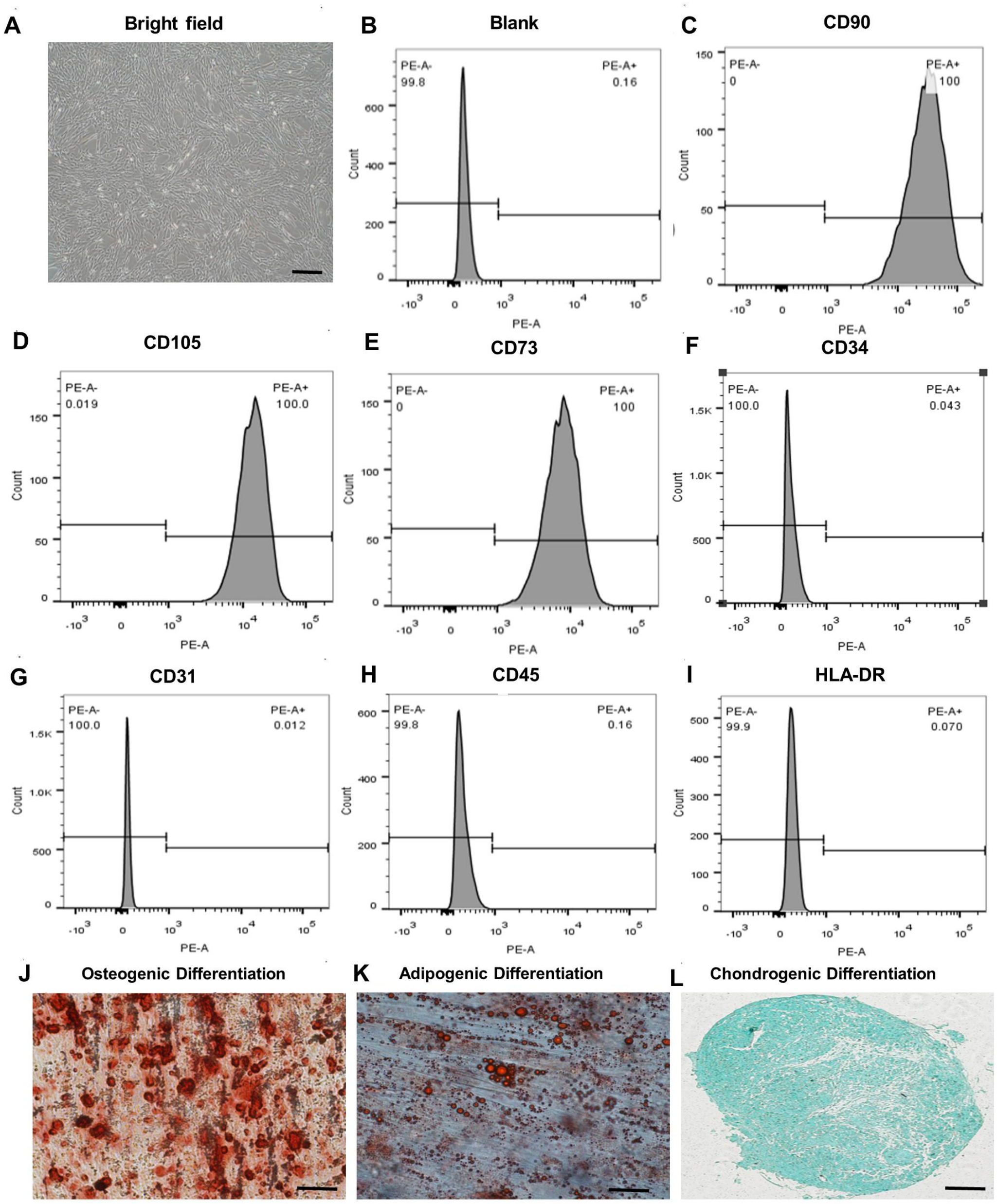

Figure S1. **Characteristics and differentiation potential of human UC-MSCs. (A)** Bright-field micrographs of UC-MSCs (P6). Scale bar = 100 μm. **(B-I)** Flow cytometry analysis for cell surface markers. The UC-MSCs were positive for CD90, CD105, and CD73, and negative for CD34, CD31, HLA-DR, and CD45. **(J-L)** Representative images of UC-MSCs differentiation into osteocytes, adipocytes, and chondrocytes. Osteogenic calcium deposition was monitored by Alizarin red staining.

Fat droplets were stained with Oil red O. Chondrogenesis was confirmed by Alcian blue staining. Scale bars = 20 μm.

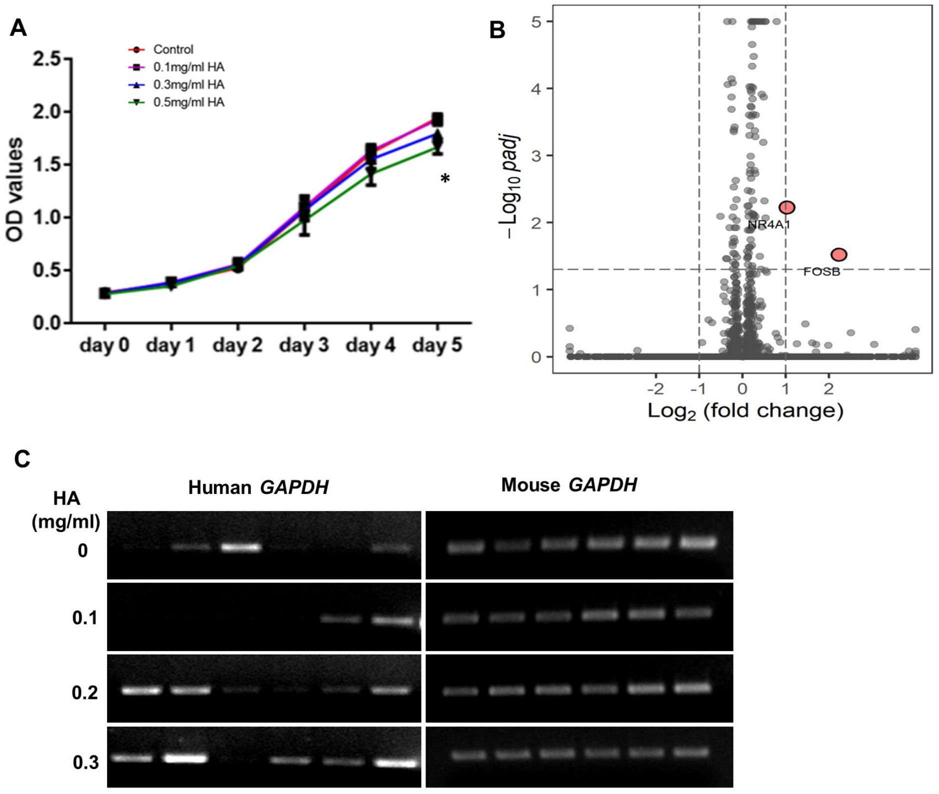

Figure S2. **Properties of UC-MSCs combined with HA *in vitro* and *in vivo*. (A)** UC-MSCs were cultured with 0-0.5 mg/ml HA for 5 days, and the CCK8 assay was performed to determine the cell viability. **(B)** The volcano plot of the differentially expressed genes between control and HA-treated groups (n = 3 for each group). The red dots indicate the two differential expression genes *NR4A1* and *FOSB* (fold change: 2.033 and 4.70, respectively). **(C)** Different concentrations (0-0.3mg/ml) of HA were combined with UC-MSCs for transplantation into mouse ovaries. The day after transplantation, stem cell tracing was performed by PCR amplification of human *GAPDH* gene. Mouse *GAPDH* gene was used as the internal control.

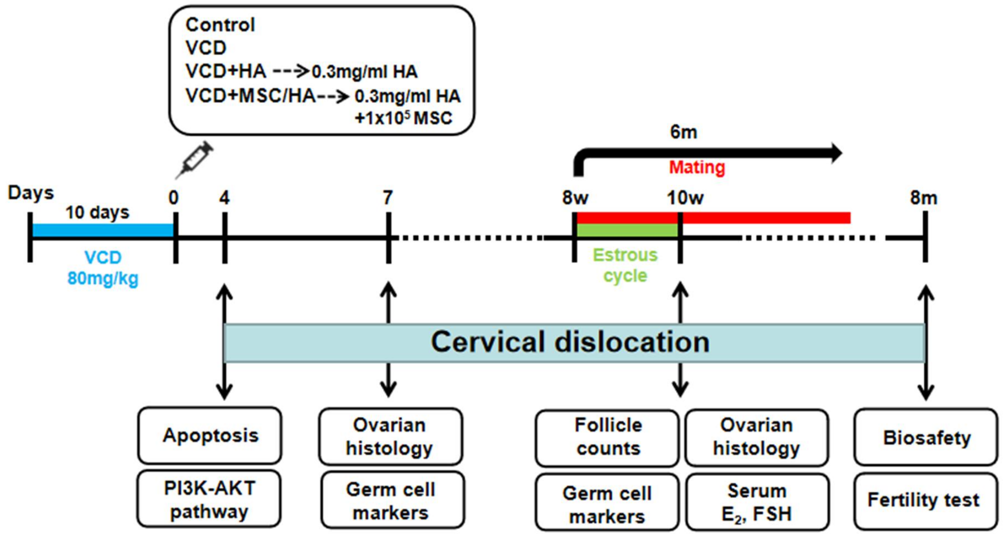

Figure S3. A diagram illustrating the four groups and the chronological order exploring the effects and mechanisms of MSC/HA transplantation in VCD-induced POI mice.

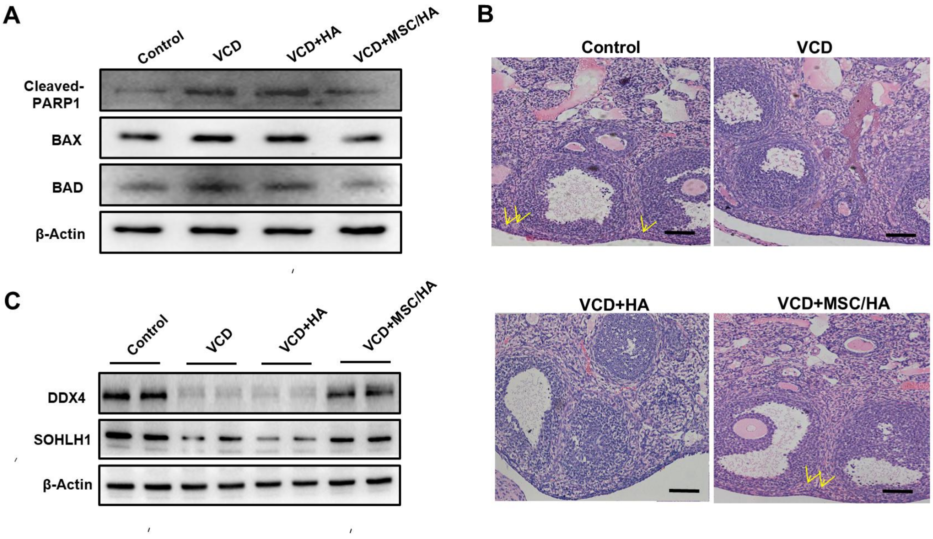

Figure S4. **MSC/HA transplantation protected against VCD-induced follicle loss and alleviated cell apoptosis *in vivo*. (A)** Western blot showing the expression of the apoptosis markers cleaved PARP1, BAX, and BAD in the four groups at 4 days after transplantation. **(B)** Histological analysis of mouse ovaries in the four groups at 1 week after transplantation. Yellow arrows indicate primordial follicles located in the ovarian cortex. Scale bar = 50 μm. **(C)** Germ cell markers DDX4 and SOHLH1 were detected by western blot at 1 week after transplantation in the four groups.

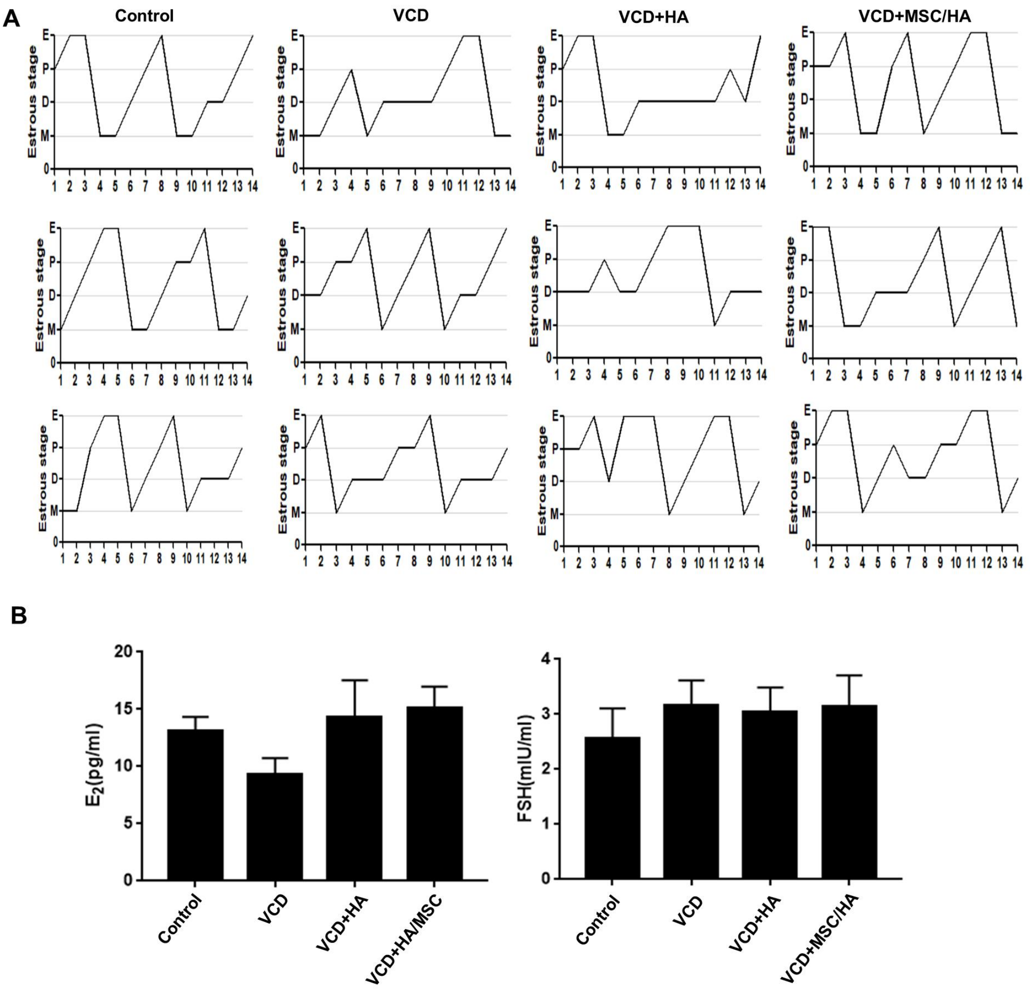

Figure S5. **Estrous cycles and hormone profiles in VCD-induced POI mice after transplantation. (A)** Vaginal smearing was used to track the estrous cycle, including the diestrus (D), proestrus (P), estrus (E), and metestrus (M) stages in the four groups at 8 week after transplantation. The mice in each group had regular estrous cycles (n = 5 for each group). **(B)** Serum levels of E_2 and FSH in the four groups were analyzed by ELISA at 10 weeks after transplantation (n = 8 for each group).

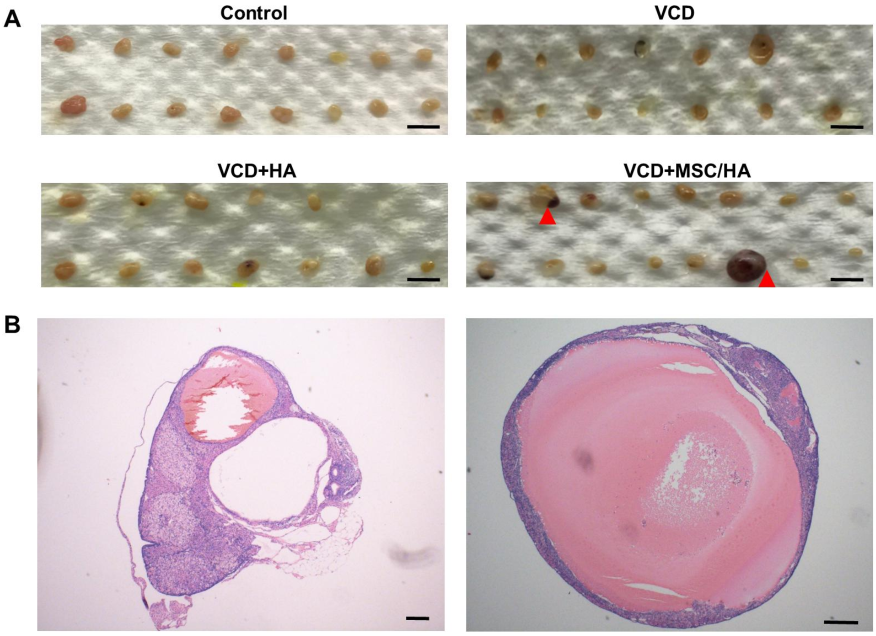

Figure S6. **Safety assessment in the ovaries after fertility test. (A)** Macroscopical images of the ovaries in the control, VCD, VCD+HA, and VCD+MSC/HA groups (n = 7 or 8 for each group). The red arrow points to ovaries needing further examination. Scale bars = 5mm. **(B)** The H&E staining of the indicated ovaries showing hematoma instead of tumor. Scale bars = 200 µm.

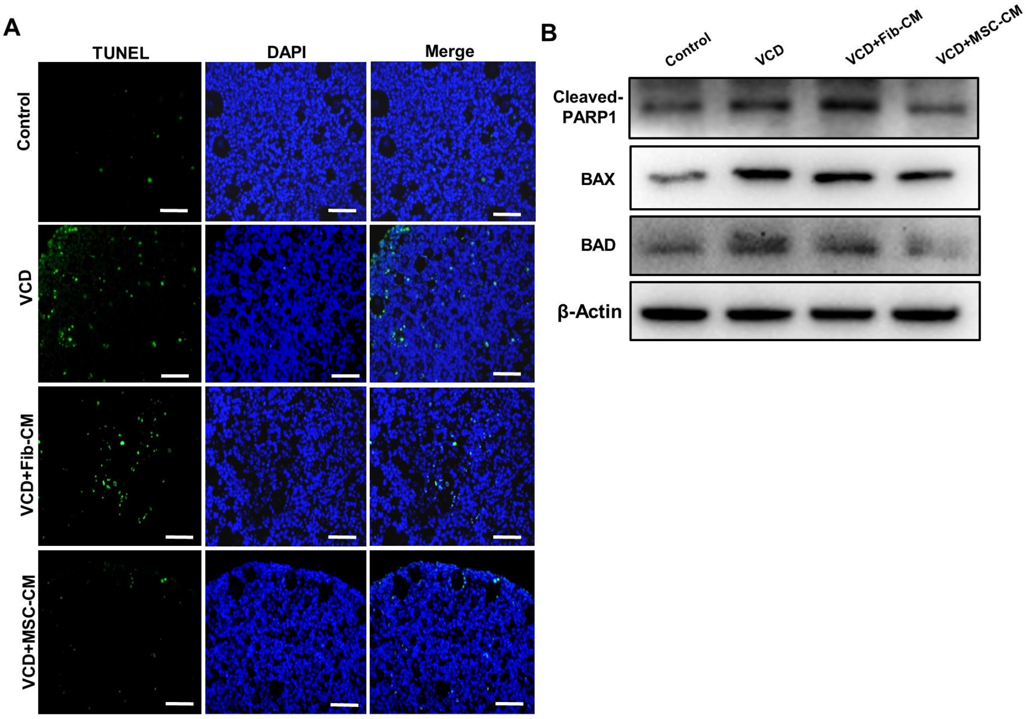

Figure S7. **MSC-CM alleviated ovarian cell apoptosis induced by VCD. (A)** Apoptosis in the ovaries of the control, VCD, VCD+Fib-CM, and VCD+MSC-CM groups was measured by TUNEL assay after 8 days of culture. The cell nuclei were counterstained with DAPI. Scale bars = 50 μm. **(B)** Western blot showing the expression of proteins related to apoptosis such as cleaved-PARP1, BAD, and BAX in the four groups after 8 days of culture.

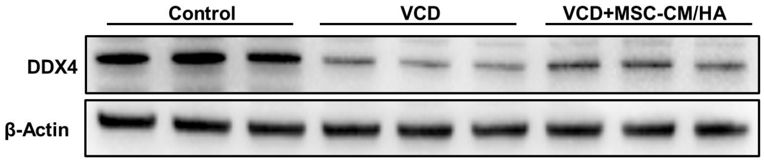

Figure S8. MSC-CM/HA was injected into the ovaries of VCD-induced POI mice, and 1 week after transplantation western blot showed the expression of DDX4 in the ovaries of the control, VCD, and MSC-CM/HA groups.

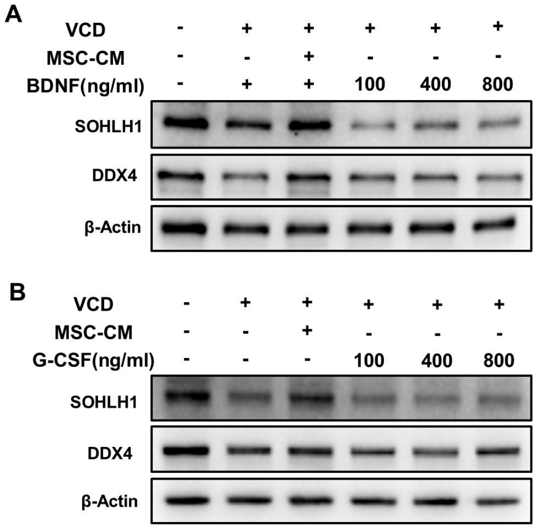

Figure S9. **BDNF and G-CSF were added to the *in vitro* ovarian culture system to determine their protective effects against VCD-induced damage.** Different concentrations of BDNF **(A)** or G-CSF **(B)** did not increase the expression of the germ cell markers DDX4 and SOHLH1 in the ovaries after 8 days of culture.

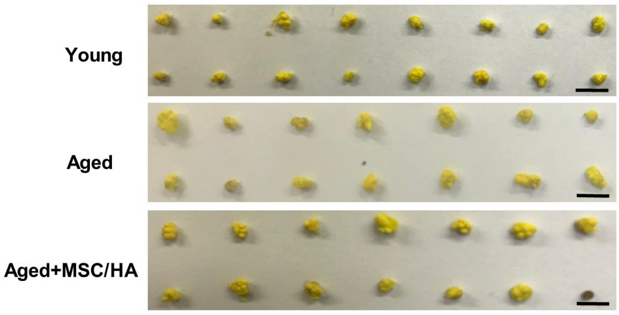

Figure S10. **Safety assessment in the ovaries of aged mice after stem cell transplantation.** Ovaries in the young, aged, and aged+MSC/HA groups were collected after fertility testing, and the macroscopic images showed no tumorigenesis (n = 7 or 8 for each group). Scale bars = 5mm.

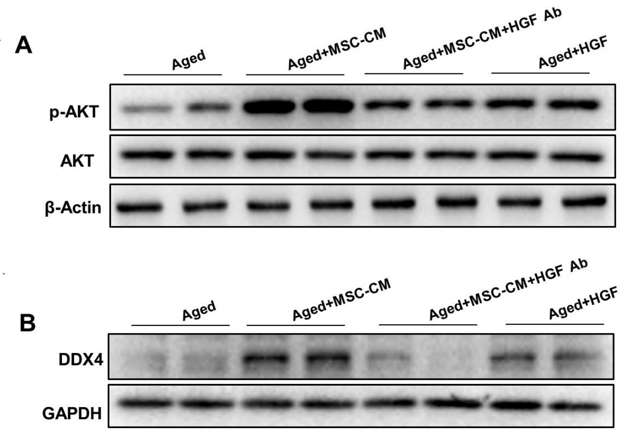

Figure S11. **HGF mediated the function of UC-MSCs in aged mice.** MSC-CM, MSC-CM with HGF antibody, and HGF were injected into the aged mouse ovaries, and the ovaries in each group were collected 4 days or 8 weeks after injection and subjected to western blot analysis. **(A)** Western blot showing the expression of p-AKT and **(B)** DDX4 after transplantation in each group.
